# Evaluation of ethanol and EDTA concentrations in the expression of biofilm-producing *smf-1*, *rpfF* genes in XDR clinical isolates of *Stenotrophomonas maltophilia*

**DOI:** 10.1186/s12866-023-03008-3

**Published:** 2023-09-30

**Authors:** Mohadeseh Ostovari Deilamani, Farhad Nikkhahi, Mehdi Bakht, Safar Ali Alizadeh, Fatemeh Fardsanei, Amir Javadi, Seyed Mahmoud Amin Marashi, Masoumeh Aslanimehr, Amir Peymani

**Affiliations:** 1 Medical Microbiology Research Center, Qazvin University of Medical Sciences, PO Box: 34199-15315, Qazvin, Iran; 2 Student research committee, Qazvin University of Medical Sciences, Qazvin, Iran

**Keywords:** Nosocomial infection, Biofilm, *Stenotrophomonas maltophilia*, Clinical isolates, EDTA, Ethanol, Checkerboard method

## Abstract

**Background:**

*Stenotrophomonas maltophilia* is able to cause infections in immunocompromised patients, and the treatment of this opportunistic pathogen is complicated due to its virulence factors, antibiotic resistance, and the ability of the bacteria to produce biofilm. The main goals of this study were to assess the susceptibility of extensively drug-resistant (XDR) isolates to ethanol and EDTA, and evaluating the synergistic effect of these disinfectants, and also survey the effect of exposure to sub-inhibitory concentrations of ethanol and EDTA on the expression of biofilm-producing *smf-1*, *rpfF* genes.

**Results:**

The results showed that EDTA significantly increased the effectiveness of the ethanol and have a synergistic effect. All of the 10 XDR isolates included in the current study harbored *smf-1* and *rpfF* genes and produced biofilm. After exposure to MIC, sub-MIC, synergism, and sub-synergism of ethanol and EDTA, the expression of *smf-1* and *rpfF* genes was repressed significantly.

**Conclusion:**

In the current study, it was indicated that the expression of biofilm-producing genes was repressed when bacteria are exposed to different concentrations of ethanol and EDTA. Future studies should include more complex microbial communities residing in the hospitals, and more disinfectants use in hospitals. Expression of other virulence genes in different conditions is suggested.

**Supplementary Information:**

The online version contains supplementary material available at 10.1186/s12866-023-03008-3.

## Background

*Stenotrophomonas maltophilia* is known as a bacterium that has recently gained importance as a nosocomial opportunistic pathogen [1]. This bacterium is associated with a variety of hospital-acquired infections such as bacteremia, pneumonia, respiratory and urinary tract infections, endocarditis, wounds, and soft tissue infections in immunocompromised patients. Acquiring resistance of *S*. *maltophilia* to antimicrobial agents and biocides is related to transposons, integrons, and plasmids [2, 3].

*S. maltophilia* is able to adhere to medical devices surfaces (catheters and respiratory therapy equipment) and epithelial mucous tissues via biofilm formation which is known as one of the important virulence factors in this bacterium [4]. It was reported that biofilms are related to 65% of acquired nosocomial infections [5]. Biofilm protects bacteria from the host’s immune system and drugs. These bacteria protect themselves by establishing a stable environment, reducing metabolism, delaying penetration into the biofilm matrix, and inducing the expression of specific proteins [4, 6]. Biofilm-producing bacteria are also resistant to nutrient deprivation, pH changes, and exposure to oxygen radicals. Biofilm-related cells grow slower than planktonic cells, and they induce changes in their gene expression, including genes that regulate osmosis and genes required for exopolysaccharide production [4, 6, 7]. Gene transfer between the bacteria in the biofilm environment is easy and as a result, will increase the number of recombinant strains. In addition, biofilm cells are up to 1000 times more resistant to antimicrobial agents [4]. Several genes are involved in biofilm development in *S. maltophilia*, including *smf-1* (*S. maltophilia* fimbriae 1) and *rpfF* (cis-11-methyl-2-dodecenoic acid) [8]. The *rpfF* gene is responsible for encoding diffusible signal factor (DSF), which regulates the expression of virulence genes such as extracellular proteases, LPS movement, and biofilm production. Cis-11-methyl-2-dodecenoic acid (RPFF) protein has several amino acid sequences similar to anoyl coenzyme A hydratase [9]. Fimbriae of *S. maltophilia* (*smf-1*) cause initial attachment to epithelial cells and participate in the early stages of biofilm formation. The gene (*smf-1*) is responsible for coding fimbriae type 1. Only isolates containing *smf-1* have the ability to form biofilms [4]. For these reason, in this study, the effect of ethanol 70%, EDTA 17%, and the mixture of different concentrations of ethanol + EDTA on the expression of biofilm-producing genes (*rpfF* and *smf-1*) in *S. maltophilia* strains was studied. The synergistic effect of ethanol and EDTA was assessed by the FIC index. Biofilm formation was assessed in sub-inhibitory concentrations of ethanol and EDTA, and their synergism effect against biofilm was determined.

One of the important causes of the spread of hospital infections is the incorrect use of disinfectants, which results in the appearance of resistant bacterial strains in hospitals. As a result, all of the medical devices and equipment used by patients can be contaminated, subsequently causing numerous infections in patients, and the length of their hospitalization [1, 10]. Over time the effectiveness of many disinfectants reduced against hospital microorganisms due to their physical and chemical structure, inappropriate use, and the lack of standardized effective concentrations [1, 10, 11]. Many studies have indicated that when bacteria are exposed to sub-inhibitory concentrations of biocides, it can lead to resistance to disinfectants and may also result in resistance to other antimicrobials [1, 10, 12]. *Pseudomonas aeruginosa*, *Acinetobacter baumannii*, and *S. maltophilia* are among the most common non-fermenting gram-negative bacilli involved in hospital infections [13, 14]. For these reason, in the current study the minimum inhibitory concentration (MIC) and minimum bactericidal concentration (MBC) of ethanol and EDTA by broth macro dilution and microplate method were determined.

Multi-drug-resistant (MDR) or extensively drug-resistant (XDR) strains are a global issue. MDR strains can be defined as non-susceptibility to at least one antibiotic in ≥ 3 antimicrobial categories and XDR is defined as non-susceptibility to at least one antibiotic in all categories but sensitive to ≤ 2 antimicrobial categories [10, 15]. Recently, Ethylene-diamine-tetra acetic acid (EDTA) has been approved as an antimicrobial agent to reduce bacterial biofilm formation. EDTA is known as a metal chelator and disrupts the outer lipopolysaccharide layer of gram-negative bacteria, metal chelators such as EDTA cause lysis and increase sensitivity to antimicrobial agents in the planktonic form of bacteria [16]. Ethanol is among the alcohols used for disinfection. Its appropriate bactericidal concentration is 60–90%. In 70% concentration, it is used as a disinfectant for devices and surfaces for 10 min. In a concentration of 100% − 90% or less than 70%, it has a lesser effect than a concentration of 70%. Disinfection of devices such as oral and rectal thermometers, laryngoscopes, laboratory desks, external surfaces of devices and equipment such as ventilators, suction, medical pressure gauges can be done with this disinfectant [17]. Alcohol-based disinfectants are also available, which is a combination of alcohol or alcohol solution and another agent (hexachlorophene, quaternary ammonium compounds, triclosan or chlorhexidine gluconate). The important point is that alcohols have weak activity against bacterial spores, protozoan oocytes and some non-lipophilic viruses [18]. In a pig tissue carrier model that was used to study disinfectant activity, 70% ethanol and 70% isopropanol were more effective than an antimicrobial soap containing 4% chlorhexidine gluconate on the titers of an enveloped bacteriophage. Contamination with alcoholic solutions is rarely reported [18]. Some studies indicated that ethanol with the mixture of EDTA has a good effect on bacterial disinfection and inhibiting biofilm production, and it was demonstrated that EDTA has a synergistic effect with ethanol [1, 10].

## Materials and methods

### Bacterial strains

A cross-sectional study was done from April 2021 to July 2022, by approval of the Ethics Committee of Qazvin Medical University (IR.QUMS.REC.1400.478). A total of 124 samples of *S. maltophilia* were collected from 1136 clinical specimens of hospitalized patients at tertiary-care hospitals in Qazvin, Iran (Velayat, Bouali, and Ghods affiliated to Qazvin University of Medical Sciences). Isolates from urine, eye discharge, wound, blood cultures, sputum, ascites, and bronchoalveolar lavage were included in the current study. All isolates were detected based on cultural, morphological, and biochemical specification (catalase and oxidase tests, gram stain, oxidative or fermentative metabolism, motility, triple sugar iron agar (TSI), deoxyribonuclease test agar (DNase), methyl red test, voges proskauer test, lysine decarboxylase, urease test, and esculin hydrolysis (Merck, Germany)) according to Bergey’s manual of systemic bacteriology, Mahon and Baily and Scott (19–21). All *S. maltophilia* isolates cultured in trypticase soy broth (TSB) then were supplemented with 10 − 15% glycerol and saved at -20 °C for further surveys. All bacterial culture media were purchased from Merck, Darmstadt, Germany. For genomic surveys, all isolates were cultured and DNA was extracted from a single colony by Kit Roche company (Germany, Lot. No.21,538,900). All *S. maltophilia* isolates were confirmed by PCR with specific 23* S rRNA* gene primers (Table 1). In the current study *S. maltophilia* ATCC 13,637, and *P. aeruginosa* ATCC 27,853 were used as the quality control strains.

**Table 1:** List of primers used in the study

Gene name	Primer Sequence (5ʹ→3ʹ)	PCR product	Annealing temperature
***smf-1***	F = ACAGGTGAGACGCAAGGA R = CAGAGCGGCAATGAGGTT	125	60.7
***rpfF***	F = AGGAAGGCGTGTTGATGG R = CTGGCGGTGTAGAGGTTG	139	60
***23 S rRNA***	F = AGAGCAGCCATAGAAGGT R = TATCGGTCGGTCAGTAGTATT	136	60

### Antibiotic susceptibility testing

Antimicrobial susceptibility testing (AST) of isolates was performed based on the Clinical and Laboratory Standards Institute (CLSI 2022) on Mueller–Hinton agar [22]. Antibiotic discs based on the CLSI 2022 were chosen and included in the current study: Meropenem (10 mg), Trimethoprim/Sulfamethoxazole (1.25/23.75 mg), Levofloxacin (5 mg), Ampicillin/Sulbactam (10/10 µg), Piperacillin/Tazobactam (PTZ, 100 /10 µg), and Minocycline (30 mg) (Mast Group Ltd., UK) which was determined using Kirby-Bauer disc diffusion method supplementary Fig. 1 (S1). The MICs for Ceftazidime and Chloramphenicol were determined using the E-test method (S2) based on CLSI 2022.

### Determining the MIC and MBC of ethanol and EDTA by broth macro dilution method

To determine the MIC of ethanol and EDTA, the broth macro dilution method was used according to CLSI 2022 [22] instructions. Firstly, a bacterial suspension was prepared with a concentration equivalent to 0.5 McFarland. For this purpose, fresh colonies (18–24 h on tryptic soy agar medium) were inoculated in a tube containing 4–5 ml of sterile physiological serum and then its turbidity was compared with the turbidity of the 0.5 McFarland standard solution by visually comparing, the microbial suspension prepared was equivalent with 0.5 McFarland. The OD of this suspension at the wavelength of 600 nm should be about 0.08 to 0.13. Then it was diluted with sterile physiological serum at a ratio of 1:100. To conduct the test, 11 sterile tubes were used, and 1 ml of Muller Hinton broth was poured into each tube, then 1 ml of the prepared biocide solutions (ethanol or EDTA) were added to tube number 1. The contents of the tube were completely mixed by a shaker, and 1 ml was picked up from it and added to tube number 2, and this process continued until tube number 9, then 1 ml was removed from tube number 9 and discarded (preparation of serial dilution). The dilutions included 1/2, 1/4, 1/8, 1/16, 1/32, 1/64, 1/128, 1/256, 1/512, and 1/1024. The active ingredient of disinfectants is available in Table 2. Tubes 11 and 12 are positive (TSB + inoculation) and negative (TSB + antimicrobial) controls. Then, except for the negative control tube, 1 ml of bacterial suspension (0.5 McFarland diluted 1 to 100) was added to all the tubes. The tubes were placed in an incubator at 37 °C for 24 h and the results after 24 h were checked visually in terms of microbial growth. The lowest concentration (highest dilution) of the biocide that prevents the growth of bacteria and no turbidity is observed in it, is reported as the MIC in mg/ml. For determining the MBC of disinfectants, 100 µl of 4 final clear diluted tubes were cultured on Muller Hinton agar, and after 48 h at 37° C, the dilution is considered as MBC that 99.9% of the bacteria did not grow [1, 10].

### Determining the MIC, MBC, and FICI of ethanol and EDTA by broth microdilution method

To approve the results of the MIC and MBC in the current study, the broth microdilution method was done (microtiter assay, 96-well plate) [8, 10]. For determining the synergistic effect of ethanol and EDTA checkerboard method was used [23]. A microtiter plate (96 wells) was used to prepare serial dilutions of EDTA. 70 µL of sterile physiological serum were added from well 1 to well 10. In the second step, 70 µL of 17% EDTA was added to the first well under completely sterile conditions. The volume of the first well became 140 µL (70 µL of sterile physiological serum + 70 µL of EDTA). After complete mixing, we remove 70 µL of the contents of the first well and add it to well number 2, and this process continued until well number 10. At the end, 70 µL were removed from well number 10 and discarded. Now, according to Tables 3 and 70 µL of ethanol concentration is added to each well, and finally, the bacterial suspension (0.5 McFarland diluted 1 to 10) is added to all the wells in the amount of 70 µL, and the final volume of each well is reached 210 µL (70 µL of bacterial suspension 0.5 McFarland diluted 1 to 10 + 70 µL of ethanol + 70 µL of EDTA) (S3). The microplate was placed in an incubator at 37°for 24 h, and after the incubation period, the results were read and the microplate was visually examined for bacterial growth (turbidity). To observe the growth in the wells, light boxes, and reflective mirrors were used [23].

Synergy and antagonism are formally calculated in the microbiology laboratory through the fractional inhibitory concentration index (FICI). FICI is calculated to quantify the interactions between the tested disinfectants. The specific value, FIC, considers the combination of disinfectants that causes the maximum change to the MIC and is calculated according to the following Eq. (24): FIC Index (EDTA & / ethanol) = FIC_A_ + FIC_B_, FIC_A_ = MIC_A_+_B_ / MIC_A_, FIC_B_ = MIC_B_+_A_ / MIC_B_.

The antagonistic effect is reported when (FIC of > 4), or addition when (FIC > 0.5 < 1), or synergistic effect when (FIC of ≤ 0.5), and indifference (FIC 1–4).

**Table 2:** Dilutions and effective ingredients of disinfectants in the present study

Well or tube number	1	2	3	4	5	6	7	8	9
Dilution	1/2	1/4	1/8	1/16	1/32	1/64	1/128	1/256	1/512
ethanol 96(%)	24	12	6	3	1.5	0.75	0.375	0.18	0.09
EDTA 17(%)	4.25	2.12	1.06	0.53	0.26	0.13	0.06	0.03	0.015

**Table 3:** Concentrations of ethanol and EDTA mixed in each well to determine FICI

	1	2	3	4	5	6	7	8	9	10
**A**	4.25 / 12	2.12 / 12	1.06 / 12	0.53 / 12	0.26 / 2	0.13 / 12	0.06 / 12	0.03 / 12	0.01 / 12	0.008 / 12
**B**	4.25 / 6	2.13 / 6	1.06 / 6	0.53 / 6	0.26 / 6	0.13 / 6	0.06 / 6	0.03 / 6	0.01 / 6	0.008 / 6
**C**	4.25 / 3	2.13 / 3	1.06 / 3	0.53 / 3	0.26 / 3	0.13 / 3	0.06 / 3	0.03 / 3	0.01 / 3	0.008 / 3
**D**	4.25 / 1.5	2.13 / 1.5	1.06 / 1.5	0.53 / 1.5	0.26 1.5	0.13 / 1.5	0.06 / 1.5	0.03 / 1.5	0.01 / 1.5	0.008 / 1.5
**E**	4.25 /0.75	2.13/ 0.75	1.06 /0.75	0.53 / 0.75	0.26/0.75	0.13 / 0.75	0.06 / 0.75	0.03 / 0.75	0.01 / 0.75	0.008 / 0.75
**F**	4.25 /0.37	2.13/ 0.37	1.06 / 0.37	0.53 / 0.37	0.26 /0.37	0.13 / 0.37	0.06 / 0.37	0.03 / 0.37	0.01 / 0.37	0.008 / 0.37
**G**	4.25 /0.18	2.13/ 0.18	1.06 / 0.18	0.53 / 0.18	0.26 /0.18	0.13 / 0.18	0.06 / 0.18	0.03 / 0.18	0.01 / 0.18	0.008 / 0.18
**H**	4.25 /0.09	2.13/ 0.09	1.06 / 0.09	0.53 / 0.09	0.26 /0.09	0.13 / 0.09	0.06 / 0.09	0.03 / 0.09	0.01 / 0.09	0.008 / 0.09

Data was express A/B (A) means Concentrations % EDTA, (B) means Ethanol Concentration %

### Assessment of biofilm formation capacity

We determined the biofilm formation ability of isolates by the crystal violet staining method in triplicates and repeated three times for each isolate (S4), based on the methods previously described [1, 10]. Cultivation of confirmed isolates of *S. maltophilia* was carried out on tryptic soy agar medium, and the isolates were incubated for 24 h in a 37 °C incubator. 2–3 colonies of fresh bacterial culture were cultured in 5 ml of sterile TSB medium in a falcon tube and incubated for 18–24 h at 37 ℃ with a shaker at 120 rpm. After 18–24 h, the optical density (OD) of all samples was read by a spectrophotometer at a wavelength of 600 nm. The concentration required to perform this test is 0.5 McFarland (1.5 × 10^8^ CFU mL − 1). 200 µL of each strain of bacteria along with the positive control (a strong biofilm-producing strain of *P. aeruginosa*) and the negative control (TSB without bacteria) were inoculated into the sterile 96-well flat-bottomed microplates and incubated in a 37 ℃ incubator for 24 h. Then the media were removed and washed three times with phosphate buffered saline (PBS: pH 7.2). In the next stage, the biofilm-producing cells attached to the plate were fixed with methanol solution 95% (200 µL/well) and fixed at room temperature (RT) for 15 min. Then, we added 200 µL of 1% crystal violet to each well and left the plate for 15 min at RT for staining. After discarding the dye, the wells were washed three times with PBS solution. In the last step, we added 200 µL of 33% acetic acid to each well and placed it on a shaker at RT for 15 min to release the biofilm formed at the bottom of the wells. The optical absorbance was measured at 570 nm (OD570, OD_C_570) using a microtiter plate reader (BioTek, Epoch, USA). Biofilm formation was categorized into 4 groups using the following formulas: If OD < ODc, the biofilm formation was negative, If ODc < OD < 2xODc, the biofilm formation was weak, if 2xODc < OD < 4xODc, the biofilm was moderate and 4xODc < OD, the biofilm was considered as strong (ODc = OD control).

### Biofilm formation in sub-inhibitory concentrations of ethanol and EDTA, and determining their synergism effect

It should be noted that, to assess biofilm formation in sub-inhibitory concentrations of ethanol and EDTA, we inoculated 150 µL of each concentration below the MIC of the disinfectant in wells. Then we added to each well 150 µL of bacterial suspension equivalent to the turbidity of 0.5 McFarland (diluted 1 to 100). For biofilm measurement in concentrations lower than synergism, we poured into each well 100 µL of ethanol, and 100 µL of EDTA in lower concentrations (sub-synergism), and 100 µL of bacterial suspension equivalent to the turbidity of 0.5 McFarland (diluted 1 to 10). We conducted it in triplicate along with the positive control (*P. aeruginosa* strong biofilm producing strain) and the negative control (TSB without bacteria). The final volume in each well reached 300 µL. After 24 h, we washed the wells three times with 1X sterile PBS solution. The rest of the steps were performed as described above.

**Biofilm formation in cell culture plate before and after exposure to ethanol and EDTA for RNA extraction**.

To prepare the samples before exposure to disinfectants, we poured 3 ml of the microbial suspension (0.5 McFarland) from each sample into the wells of the 12-well cell culture plate (S5). To prepare samples after exposure to disinfectant, the first, second, and third MIC and sub-MIC dilutions of the disinfectant along with 0.5 McFarland’s bacteria diluted 1 to 100 poured into each well of the 12-well cell culture plate (1.5 ml of ethanol and 1.5 ml of 0.5 McFarland bacteria (diluted 1 to 100), as well as 1.5 ml EDTA and 1.5 ml 0.5 McFarland bacteria (diluted 1 to 100). To prepare samples after being exposed to two disinfectants, the first, second, and third dilutions of synergism and sub-synergism were equally poured into each well of the 12-well cell culture plate along with 0.5 McFarland bacteria (diluted 1 to 10, 1 ml of ethanol, 1 ml of EDTA, and 1 ml of 0.5 McFarland 1 diluted 10) plates were incubated at 37 °C for 24 h. Then 3 ml of 1X sterile PBS was added to each well. We scraped the bottom and all the walls of the well until the formed biofilm was completely removed, and then the content inside the well was completely transferred to the sterile RNase and DNase free microtube. Then, samples were centrifuged for 20 min at 7000 rpm. Finally, the supernatant inside the microtube was discarded and the sediment left at the bottom of the microtube was transferred to a -80 °C freezer for RNA extraction.

### Molecular method for detection of *smf-1* and *rpfF* gene

Detection of the presence of *rpfF* and *smf-1* biofilm genes in XDR isolates of *S. maltophilia* was done by PCR method; primer sequences used are available in Table 1. In order to ensure the accuracy of the PCR steps and to ensure the absence of contamination in the test in each series of PCR tests, distilled water was used as a negative control and one isolate was analyzed as a positive control along with other unknown samples. As a positive control, *S. maltophilia* strain 13,637 registered as a strain containing *rpfF* and *smf-1* genes was used [25]. The PCR reactions were done in a total volume of 25 mL containing 50 mM KCl, 0.2 mM of each dNTP, 10 mM Tris–HCl (pH 8.3), 1.5 mM MgCl2, 0.5 mM of each primer, and 1 U Taq DNA polymerase. We sequenced (23* S rRNA*) a representative amplicon of *23 S rRNA* gene was subjected to sequencing (Microsynth Switzerland company) and the sequence was deposited in GenBank and assigned the accession no MZ468054. We analyzed using BLAST (http://www.ncbi.nlm.nih.gov/BLAST/) for each gene.

### Determination of *smf-1* and *rpfF* gene expression changes before and after exposure to disinfectant by the real-time quantification polymerase chain reaction (qRT-PCR)

To extract RNA from MIC, sub-MIC, synergism, and sub-synergism concentrations the Beh gene company’s RNA extraction kit (code number BPVD050) was used. The gene expression level of the *smf-1* and *rpfF* genes was examined under different conditions: MIC, sub-MIC, synergism, and sub-synergism. cDNA synthesis (cDNA Synthesis Kit, Thermo Scientific, United States) for each isolate was performed according to the manufacturer’s instructions. The levels of expression of the genes involved in biofilm formation were measured in triplicate using SYBR Premix Ex Taq II (Takara Bio, Inc., Japan). Using the ABI Step OneTM System (Applied Biosystems, San Francisco, CA, United States), qRT-PCR was performed in a 25-ml total reaction volume containing cDNA and specific primers. RT-PCR was carried out with the following cycle profile: 1 cycle at 95°C for 30 s, followed by 40 cycles at 95°C for 5 s, 60°C for 10 s, and 72°C for 20 s. The housekeeping gene 23* S rRNA* was used as an internal control to normalize the levels of each gene transcript. The fold changes of the target gene expression levels were calculated by the 2-*11C*T method. Differentially expressions of genes were analyzed with the use of the criteria threshold of twofold change. The primary data obtained from Real-time PCR were analyzed using the threshold cycle (CT) comparative method. Then, in order to calculate the ∆Ct values of each gene, the Ct of each amplified gene was normalized with the Ct of the corresponding 23* S rRNA* gene. At the end, the ∆Ct values obtained from each sample were compared with the ∆Ct values obtained for the bacterial control sample (the sample before exposure). The ∆Ct values obtained from each of the MIC and sub-MIC concentrations as well as the synergism and sub-synergism concentrations of ethanol and EDTA were compared with the ∆Ct values obtained for the control sample (sample before exposure) using the t-test method [26, 27]. Then these differences were assessed by Student’s *t*-test for consideration as statistically significant. A *P*-value of ≤ 0.05 was considered a significant level. The primer sequences used in the qRT-PCR are described in Table 1.

## Results

### Description of clinical isolates

**Fig. 1:**
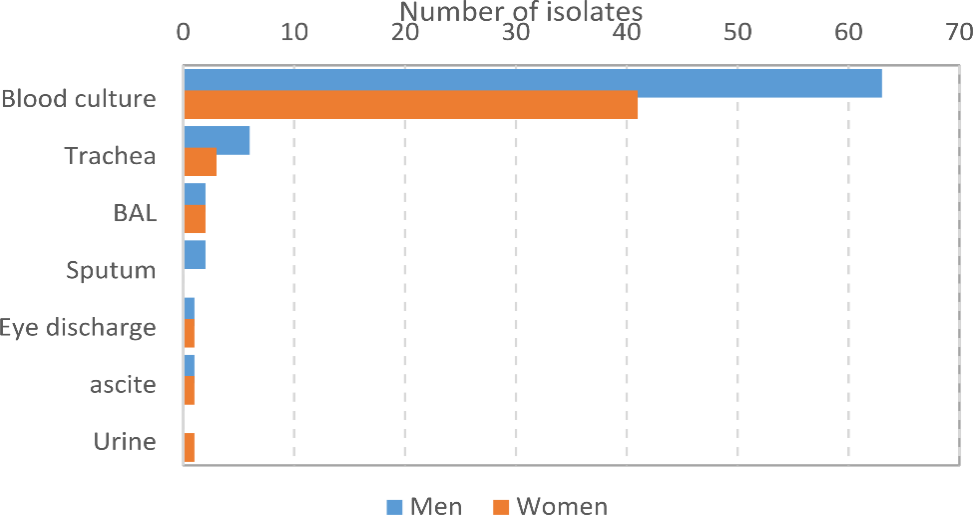
Diagram of occurrence of *S. maltophilia* in relation to clinical source

**Fig. 2:**
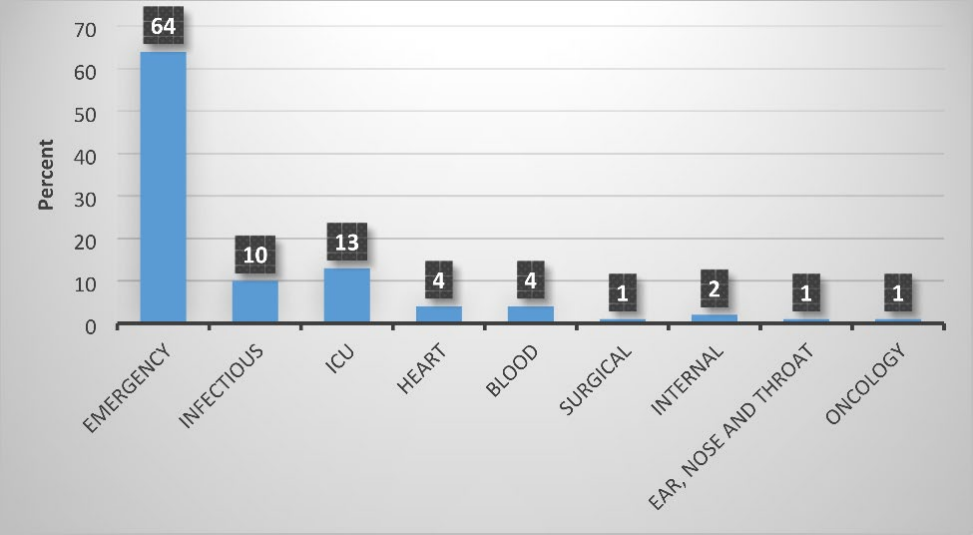
Diagram of occurrence of *S. maltophilia* in relation to hospital department source

Biochemical tests and the presence of the 23* S rRNA* gene confirmed their identity as *S. maltophilia*. *S. maltophilia* strain 13,637 was used as a positive control for 23* S rRNA*. During this research, 124 isolates of *S. maltophilia* were obtained from hospitalized patients, of which 75 samples (60.5%) belonged to men and 49 samples (39.5%) belonged to women. Also, the age distribution of people (2 days − 85 years old) showed that 9 samples belonged to children, 3 of which were isolated from infants less than 1-month-old. Out of the total number of isolates, the highest number of isolates were from blood culture (104 isolates) and the least number of isolates were isolated from the urine sample (1 isolate). Also, the highest frequency of samples was related to the emergency department with 79 samples (63.8%), and the lowest frequency was related to the departments of ear, nose and throat, and oncology, each with 1 sample (0.8%). The information about the type of sample and gender of the patients is shown in Fig. 1 and the inpatient department in Fig. 2.

### Antimicrobial susceptibility testing

**Fig. 3:**
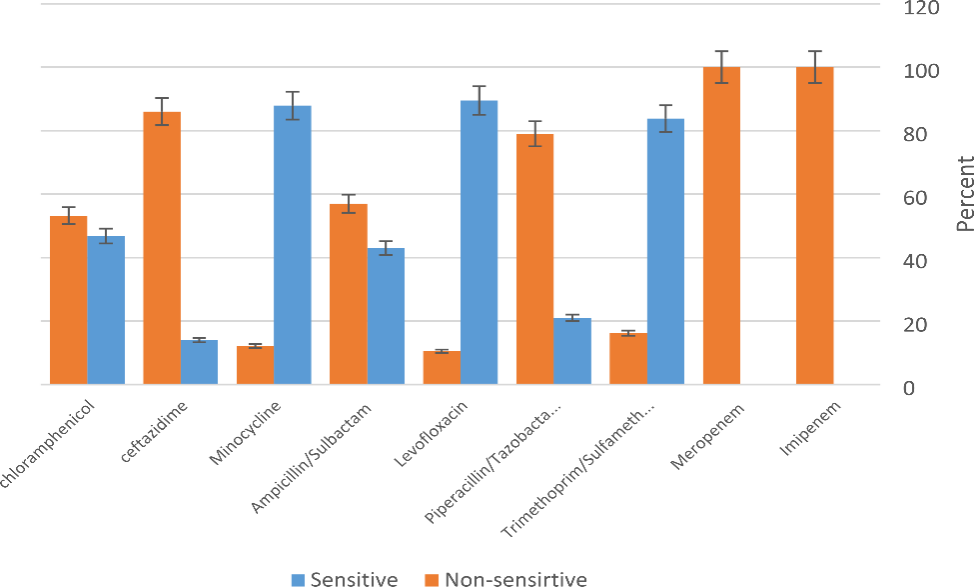
Diagram of the results of antibiotics susceptibility test

The antimicrobial susceptibility patterns of the 124 isolates are shown in Fig. 3. Of the 124 isolates, 40 (32.2%) were MDR and 10 (8%) isolates were XDR according to CLSI 2022 [22]. According to the goals of this study, 10 XDR samples entered the later stages of the study, and 114 other isolates were excluded from the study.

### Determination of MIC and MBC of ethanol and EDTA by microbroth dilution method

In this study, the most effective concentration of ethanol was 1/8 (6%) which is determined as MIC and MBC ethanol (Table 4). The MIC for EDTA was 1/128 (0.06%), but EDTA was not able to kill isolates, and MBC was not determined.

**Table 4:** MIC and MBC of ethanol and EDTA by microbroth dilution method

Well number	1	2	3	4	5	6	7	8	9	10	11
Serial dilution	1/2	1/4	1/8	1/16	1/32	1/64	1/128	1/256	1/512	Control +	Control -
ethanol 96%	24%	12%	6%	3%	1.5%	0.75%	%0.375	0.18%	0.09%		
MIC	-	-	-	+	+	+	+	+	+	+	-
MBC	-	-	-	+	+	+	+	+	+	+	-
EDTA17%	4.25	2.125	1.06	0.53	0.26	0.13	0.06	0.03	0.015	Control +	Control -
MIC	-	-	-	-	-	-	-	+	+	+	-
MBC	+	+	+	+	+	+	+	+	+	-	-

### Investigating the synergistic effect of ethanol and EDTA using the checkerboard method

According to Table 3, different concentrations of ethanol and EDTA were mixed in each well, and after 24 h of incubation at 37℃, the results of microbial growth were determined (Table 5). FICI was calculated for ethanol and EDTA. Considering that the FICI is less than 0.5, therefore, in the concentration of 0.01% of EDTA and the concentration of 1.5% of ethanol, these two substances have a synergistic effect (Table 5). In addition to the 96-well microplate, the synergism effect of ethanol and EDTA was investigated by the checkerboard method in sterile tubes and also sterile micro tubes to confirm the above results. According to the microbial growth of the plates and repeating the checkerboard results, it was found that the effect of ethanol and EDTA is not dependent on time.

**Table 5:** Calculation of ethanol and EDTA FICI using the checkerboard method

**Ethanol concentration %**		**EDTA Concentration %**										
			**1**	**2**	**3**	**4**	**5**	**6**	**7**	**8**	**9**	**10**
			4.25	2.125	1.06	0.53	0.26	0.13	0.06	0.03	0.01	0.008
	**A**	12	-	-	-	-	-	-	-	-	-	-
	**B**	6	-	-	-	-	-	-	-	-	-	-
	**C**	3	-	-	-	-	-	-	-	-	-	-
	**D**	1.5	-	-	-	-	-	-	-	-	-	+
	**E**	0.75	-	-	-	-	-	-	-	+	+	+
	**F**	0.375	-	-	-	-	-	-	-	+	+	+
	**G**	0.18	-	-	-	-	-	-	+	+	+	+
	**H**	0.09	-	-	-	-	-	-	+	+	+	+

### **Biofilm formation assessment**

The phenotypic investigation of biofilm formation in XDR isolates before exposure, in MIC concentration, sub-inhibitory concentrations of ethanol and EDTA, as well as their synergism effect, was studied by the microtiter-plate method, and the OD of the samples obtained from the ELISA reader at a wavelength of 570 nm. According to the results obtained from this study, before the strains were exposed to ethanol and EDTA, they formed a strong biofilm. Among the 10 XDR isolates, 7 samples strongly and 3 isolates moderately formed biofilm. *S. maltophilia* 13,637 also formed a moderate biofilm. It should be noted that, when they were exposed to different concentrations of ethanol and EDTA (MIC, sub-MIC_1 − 3_), their biofilm formation power decreased. results are shown in Tables 6, 7, 8 and 9.

Optical absorbance of negative control = TSB without bacteria = 0.15.

Optical absorbance of positive control = *P. aeruginosa* strong biofilm producing strain = 3.845.

**Table 6:** Optical absorbance of isolates in biofilm formation before and after exposure to ethanol

Isolates	Before exposure to ethanol	MIC = 6%	sub-MIC1 = 12%	sub-MIC2 = 24%	sub-MIC3 = 48%
1	3.06	0.181	0.289	0.299	0.320
2	2.96	0.192	0.295	0.304	0.322
3	2.85	0.188	0.276	0.282	0.296
4	3.14	0.195	0.284	0.289	0.302
5	3.21	0.211	0.321	0.336	0.354
6	2.89	0.19	0.28	0.294	0.298
7	2.05	0.162	0.237	0.241	0.262
8	2.3	0.173	0.263	0.277	0.281
9	2.14	0.168	0.254	0.269	0.273
10	3.11	0.186	0.293	0.305	0.328

**Table 7:** OD of isolates in biofilm formation before and after exposure to EDTA

Isolates	Before exposure to EDTA	MIC =%0.06	sub-MIC1=%0.03	sub-MIC2=%0.015	sub-MIC3=%0.008
ST	2.87	0.165	0.174	0.198	0.346
1	3.06	0.175	0.18	0.208	0.364
2	2.96	0.194	0.211	0.235	0.352
3	2.85	0.168	0.177	0.196	0.338
4	3.14	0.188	0.196	0.217	0.443
5	3.21	0.191	0.236	0.255	0.471
6	2.89	0.176	0.183	0.197	0.352
7	2.05	0.131	0.143	0.166	0.289
8	2.3	0.52	0.172	0.194	0.311
9	2.14	0.151	0.164	0.183	0.302
10	3.11	0.181	0.188	0.239	0.372

**Table 8:** OD of isolates in biofilm formation at synergism and sub-synergism concentrations

Isolates	Before exposure to EDTA + ethanol	synergism concenteration = EDTA (0.01%)+ ethanol(1.5%)	sub-syn1= EDTA (0.008%)+ ethanol(1.5%)	sub-syn2= EDTA (0.008%)+ ethanol(1.5%)	sub-syn3= EDTA (0.002%)+ ethanol(1.5%)
ST	2.87	0.158	0.18	0.218	0.223
1	3.06	0.172	0.191	0.230	0.245
2	2.96	0.165	0.186	0.224	0.23
3	2.85	0.154	0.167	0.210	0.216
4	3.14	0.182	0.210	0.245	0.262
5	3.21	0.188	0.205	0.248	0.311
6	2.89	0.161	0.178	0.214	0.228
7	2.05	0.112	0.129	0.167	0.181
8	2.3	0.147	0.163	0.205	0.218
9	2.14	0.137	0.151	0.185	0.211
10	3.11	0.176	0.202	0.238	0.246

**Table 9:** OD of isolates in biofilm formation in different mixed dilutions of alcohol ethanol and EDTA

**Ethanol concentration %**	**EDTA concentration %**				
		0.06	0.03	0.015	0.008
	1.5	NG	NG	0.172	0.191
	0.75	NG	0.216	0.266	0.301
	0.375	NG	0.236	0.285	0.329
	0.18	0.249	0.263	0.298	0.507
	0.09	0.296	0.308	0.337	0.978

### Biofilm-producing genes in XDR isolates

*rpfF* and *smf-1* genes were detected using the PCR technique. Among the 10 XDR isolates, all isolates (100%) contained the *rpfF* and *smf-1* genes (S6 and S7).

### Expression changes of *smf-1* and *rpfF* genes

**Fig. 4:**
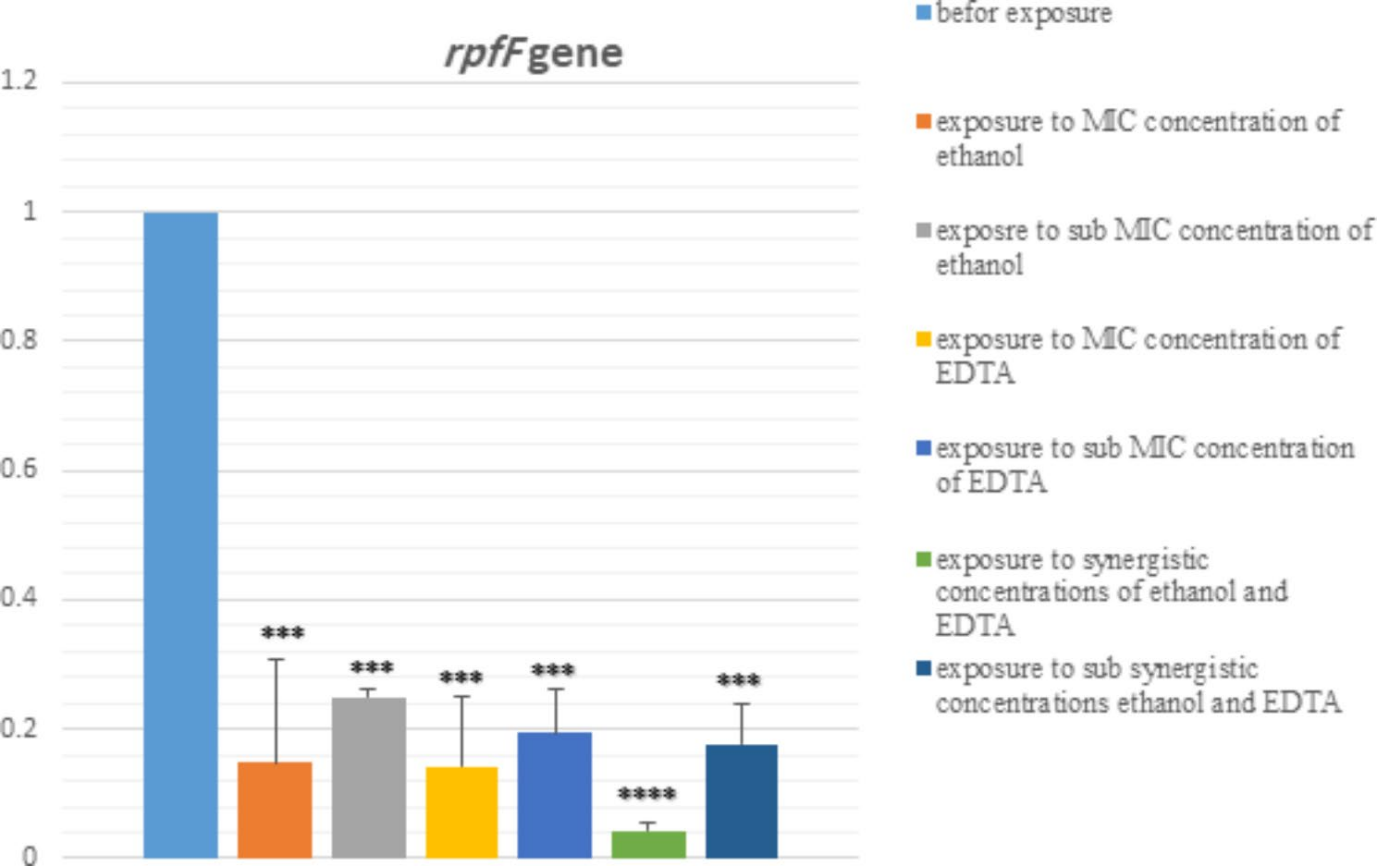
The effect of MIC and sub-MIC concentrations of ethanol and EDTA, as well as their synergism and sub- synergism concentrations on *rpfF* gene expression assessed by qRT–PCR. *P < 0.05

**Fig. 5:**
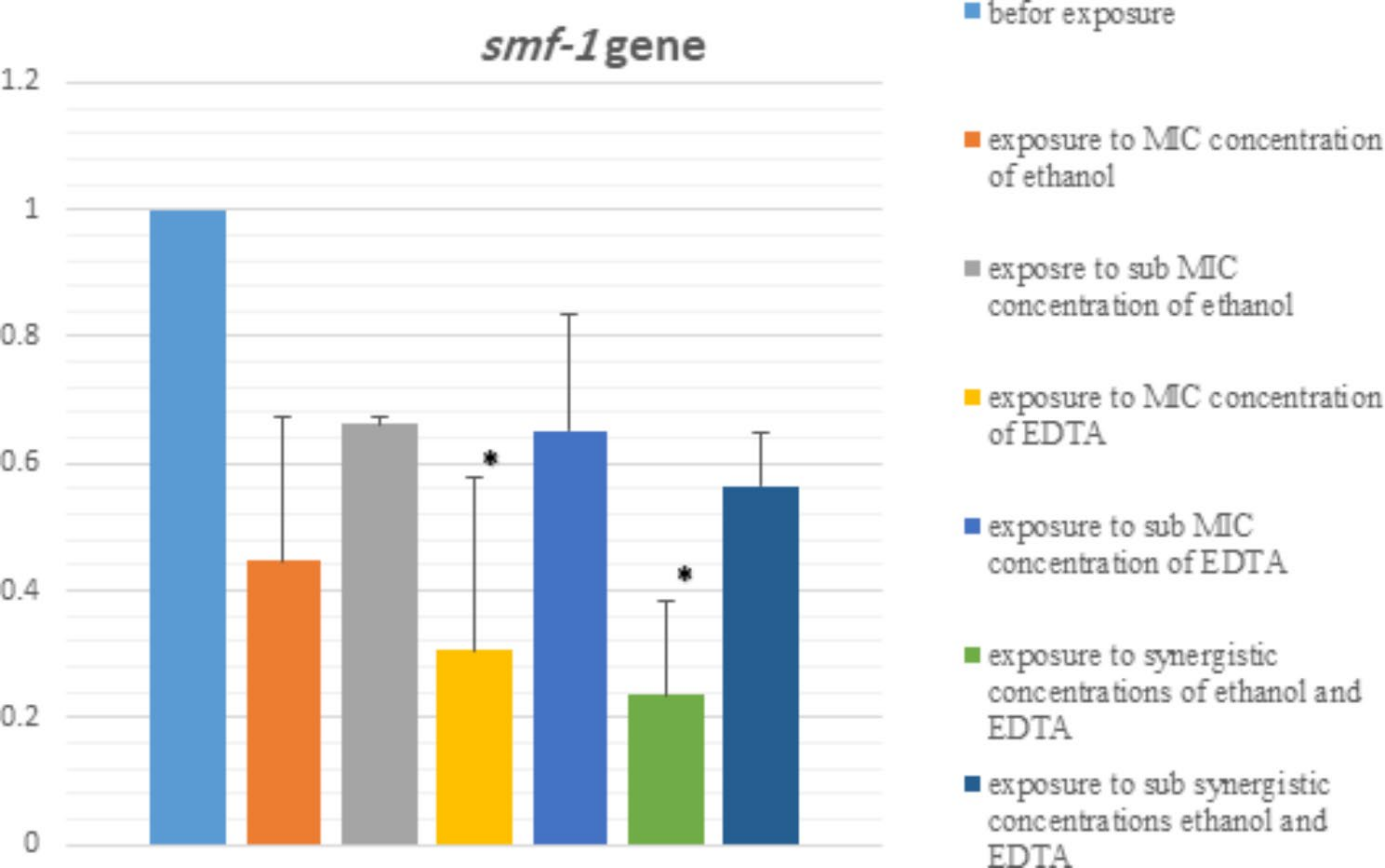
The effect of MIC and sub-MIC concentrations of ethanol and EDTA, as well as their synergism and sub- synergism concentrations on *smf-1* gene expression assessed by qRT–PCR. *P < 0.05

In Figs. 4 and 5, the effect of MIC and sub-MIC concentrations of ethanol and EDTA, their synergism and sub-synergism concentrations on the expression of *rpfF* and *smf-1* genes are shown. We observed a significant difference in the expression of *rpfF* and *smf-1* genes at different conditions (MIC, sub-MIC, synergistic, sub-synergism, and before exposure). After exposure of the strains to ethanol and EDTA, the expression of biofilm genes decreased. But the biggest decrease in expression is related to the synergistic effect of ethanol and EDTA. Based on the result, the expression of gene *rpfF* has changed more than the gene *smf-1.* Melting curve related to *rpfF* and *smf-1* genes are shown in S8 and S9.

## Discussion

An important feature of *S. maltophilia* is its ability to attach strongly and form biofilm on surfaces, water sources [28], medical implants, and catheters [7, 8], especially in cystic fibrosis patients [29]. It suggested that biofilm formation could be an important stage in colonization, and serve as a potential source of waterborne transmission of pathogens to livestock and humans [28]. Recent clinical studies prominent an increase in the frequency of isolation of *S. maltophilia* in hospitals [1, 8]. It was reported that up to 60–80% of all bacterial infections are related to biofilm formation [5, 30]. The results of studies showed that isolates that produced strong and intermediate biofilm are more resistant to antibiotics and disinfectants [1, 10]. Biofilm formation is different among the clinical isolates of *S. maltophilia*. It can be related to the presence and expression of different genes. Thus, in the current study, we assessed the association of biofilm formation and the presence and expression of *smf-1*, and *rpfF* genes.

In some studies, it was reported that biofilm formation is associated with the *smf-1* in *S. maltophilia* [8, 31].

In the current study, we selected 10 XDR *S. maltophilia*, 7 of them produced strong biofilm and 3 were intermediate biofilm producers. All of the isolates harbored *smf-1*, and *rpfF* which are biofilm-related genes that is in line with a study conducted in China [6], in which the prevalence of *rpfF* gene was reported lower than our results which can be due to sample size and selection of isolates in our study.

The expression of these genes was assessed by qRT–PCR in different conditions (before exposure, MIC, sub-MIC, synergism, and sub-synergism concentrations). Statistical comparison of the relative expressions of the genes revealed that *rpfF* was expressed in MIC, sub-MIC, synergism, and sub-synergism concentrations of ethanol and EDTA at a lower level (*P < 0.05). The lowest gene expression was related to the synergistic effect of ethanol and EDTA (Figs. 4 and 5). Also, assessment of expression of the gene *smf-1* revealed that in the concentrations of MIC, sub-MIC, synergism, and sub-synergism of ethanol and EDTA *smf-1* is expressed at a lower level. But the statistical comparison was only significant in two cases (synergistic effect of ethanol and EDTA, and after exposure to MIC concentration of EDTA) (Fig. 5).

According to the results of this study, EDTA and ethanol not only is able to disrupt the biofilm in bacteria but also is able to decrease the expression of biofilm-related genes at appropriate concentrations. Also, EDTA and ethanol have the best synergistic effect on decreasing the expression of biofilm-related genes. EDTA has been known as a sensitizing and potentiating agent. Several surveys revealed that biofilm disrupting action of EDTA is due to its ability to cations sequestering, and increases the effectiveness of other antimicrobial agents [32].

In hospitals, treatment of infection due to *S. maltophilia* is difficult because it is naturally resistant to different classes of antibiotics, as a result, it is prominent necessary to develop novel drugs and detergents to combat this bacterium [33], and other bacteria [10, 34]. In evaluating the power of biofilm formation in different dilutions of ethanol and EDTA, when the isolates were not exposed to ethanol and EDTA, they formed a strong biofilm. After exposure to different dilutions of the disinfectant, the results showed that the power of biofilm formation decreased with the increase in the concentration of the disinfectant (Tables 6, 7, 8 and 9). Results of antimicrobial susceptibility testing showed that the rate of MDR/XDR in *S. maltophilia* clinical isolates in this study is lower compared to another study conducted in China [31]. A study conducted in Iran indicated that most of the *S. maltophilia* strains produce biofilm. Also, most of them are MDR/XDR which is in line with this study. In that study it was demonstrated that EDTA has best synergistic effect on ethanol compared to the other disinfectant which is consistent with our result [1].

## Conclusions

In the current study, we indicated that EDTA has a synergistic effect with ethanol. The results which are extracted from this research, suggest to use of alternative compounds such as EDTA in combination with disinfectant to increase the potency of disinfectant by creating synergistic effects against MDR/XDR *S. maltophilia* isolates. EDTA is also fully biodegradable, less harmful to the environment and human health, and has no toxic effects on humans compared to other disinfectants. The current survey has some limitations. First, because of cost limitation, we just phenotypically assessed biofilm formation (spectrophotometric microtitre assay), and this method measures both viable and unviable bacteria. Also, other genes play a role in biofilm formation, which should be investigated in future studies, and due to financial limitations, we were not able to do it in the current study. Also, studies should be conducted on the relationship of other disinfectants (sodium hypochlorite, chlorhexidine, Dettol), which are widely used in hospitals, with inhibition of gene expression. We also planned to do typing for these isolates, but due to cost constraints, it has not been done yet. Future studies should include more complex microbial communities residing in the hospitals. Also, to the field of study using EDTA in combination with ethanol should be addressed.

### Electronic supplementary material

Below is the link to the electronic supplementary material.

Supplementary Material 1

## Data Availability

All data and materials are available upon request to corresponding author. The datasets generated and/or analysed during the current study are available in the GenBank repository Gene data: 23 S *rRNA* sequence data: GenBank accession number MZ468054.

## Declarations

### Statistical analysis

Descriptive statistics were used to measure the characteristics of the study. Pearson chi-square test was used to determine significant differences between proportion. P values of < 0.05 were considered significant. Statistical analysis was performed by using SPSS version 16.0 statistical software (SPSS Inc., Chicago, IL, USA).

## Abbreviations

MDR: Multidrug-resistant
XDR: Extensively drug-resistant
MIC: Minimum inhibitory concentration
MBC: Minimum bactericidal concentration
WHO: World Health Organization
EDTA: Ethylene-diamine-tetra acetic acid
FICI: Fractional inhibitory concentration index
DSF: Diffusible signal factor
AST: Antimicrobial susceptibility testing
CLSI: Clinical and Laboratory Standards Institute
ODc: OD control
OD: Optical density
TSB: Trypticase Soy Broth
ST: Standard
