# Supplementary material for: Evaluation of ethanol and EDTA concentrations in the expression of biofilm-producing smf-1, rpfF genes in XDR clinical isolates of Stenotrophomonas maltophilia
Source: BMC Microbiol. 2023 Sep 30;23:277. doi: 10.1186/s12866-023-03008-3 (PMC10542227; doi:10.1186/s12866-023-03008-3)
Supplement: Supplementary file 1 — Supplementary Material 1 [file 12866_2023_3008_MOESM1_ESM.docx]

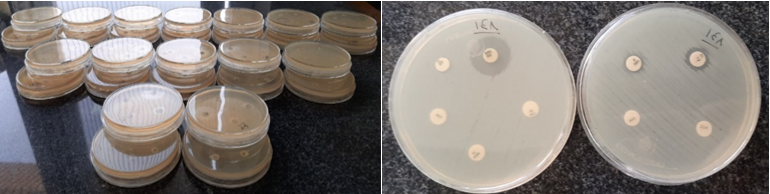


**Fig S1** Antimicrobial susceptibility testing of isolates using Kirby-Bauer disc diffusion method


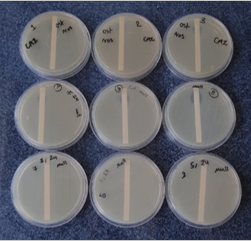


**Fig S2** The MICs for Ceftazidime and Chloramphenicol were determined using the E-test method


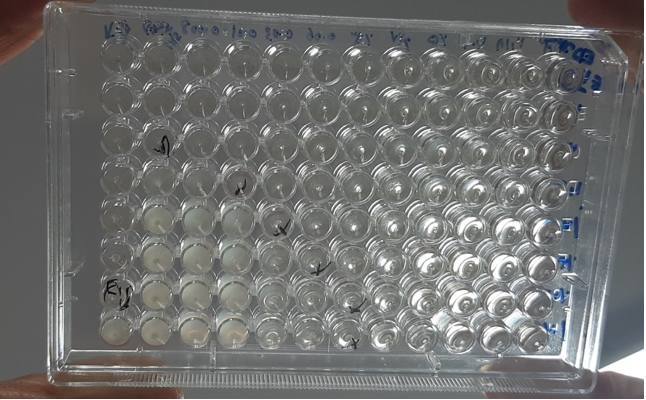


**Fig S3** Ethanol and EDTA mixed in each well to determine FICI


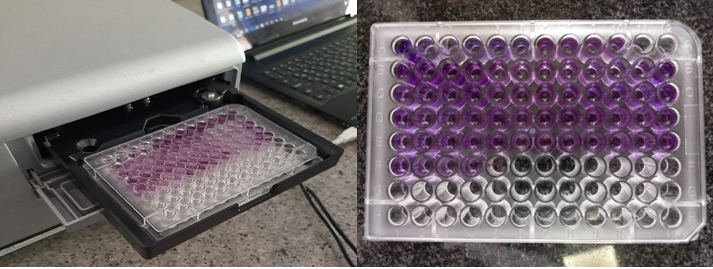


**Fig S4** Biofilm formation by microtitreplate method


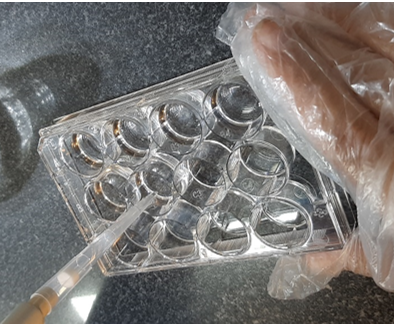


**Fig S5** Biofilm formation in 12-well cell culture plate for RNA extraction


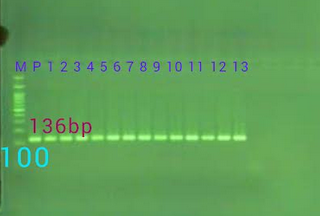


**Fig S6** Gel electrophoresis of the PCR amplified products of *rpfF* gene for the *S. maltophilia* isolates with 139 bp amplification fragment. Lane M: DNA size marker - Lane P: positive control - Lane 1–10: *rpfF* positive isolates


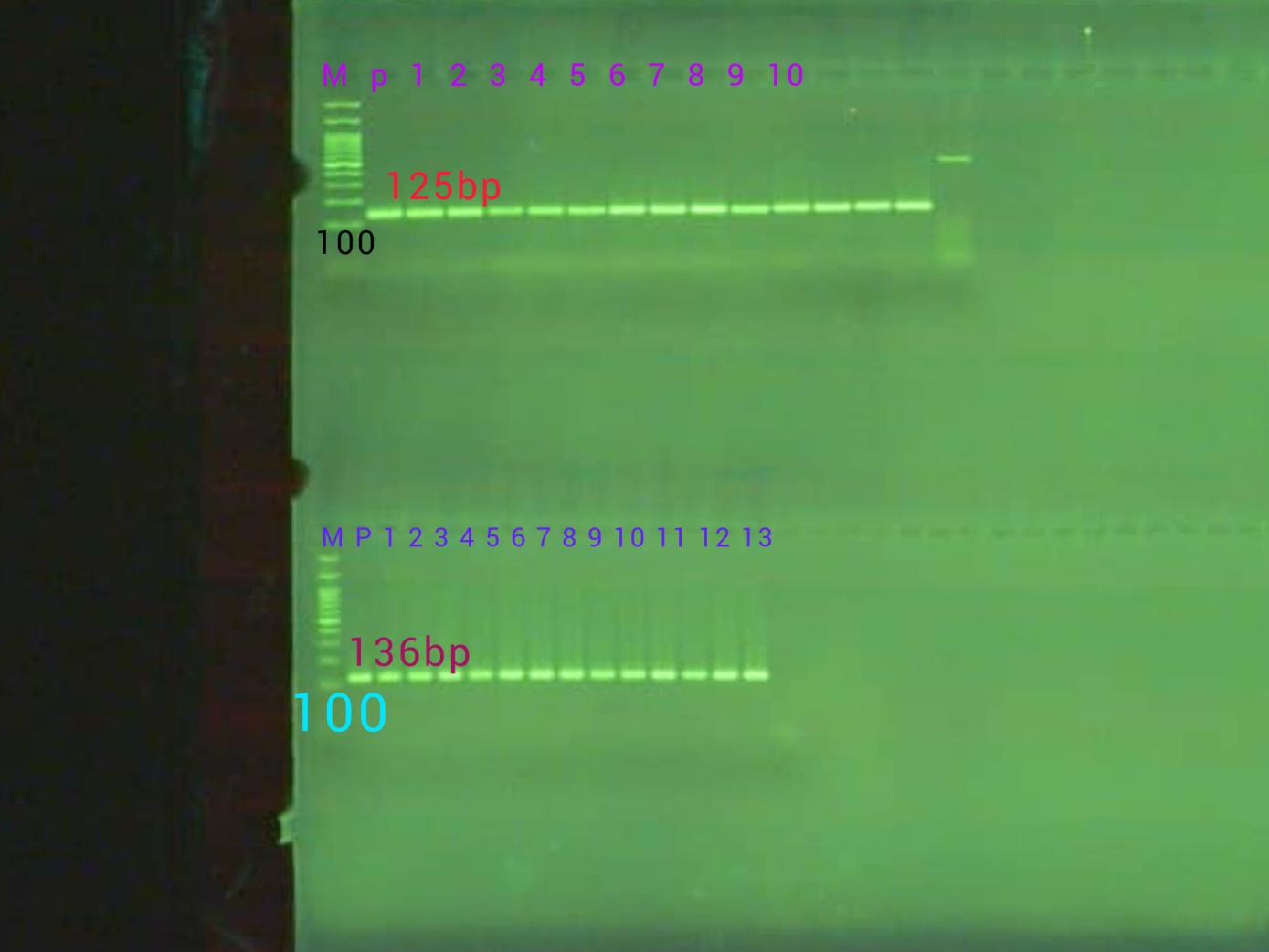


**Fig S7** Gel electrophoresis of the PCR amplified products of *smf-1* gene for the *S. maltophilia* isolates with 125bp amplification fragment. Lane M: DNA size marker - Lane P: positive control - Lane 1–10: *smf-1* positive isolates


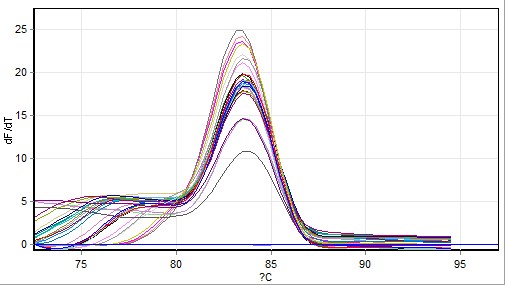


**Fig S8** Melting curve related to *rpfF* gene before and after exposure to MIC and sub-MIC concentrations of ethanol and EDTA alone and their synergism and sub-synergism concentrations.


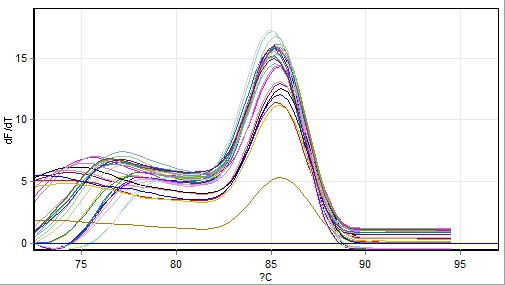


**Fig S9** Melting curve related to *smf-1* gene before and after exposure to MIC and sub-MIC concentrations of ethanol and EDTA alone and their synergism and sub-synergism concentrations.
